# Supplementary material for: Impact of Scala Tympani Geometry on Insertion Forces during Implantation
Source: Biosensors (Basel). 2022 Nov 10;12(11):999. doi: 10.3390/bios12110999 (PMC9688204; doi:10.3390/bios12110999)
Supplement: Supplementary file 1 [file biosensors-12-00999-s001.zip › biosensors-1924497-supplementary.pdf]

biosensors

MDPI

Supplementary data

Impact of Scala Tympani Geometry on Insertion Forces during Implantation

Filip Hrncirik, Iwan V. Roberts, Chloe Swords, Peter J. Christopher, Akil Chhabu, Andrew H. Gee, Manohar L. Bance*

* Correspondence: mlb59@cam.ac.uk

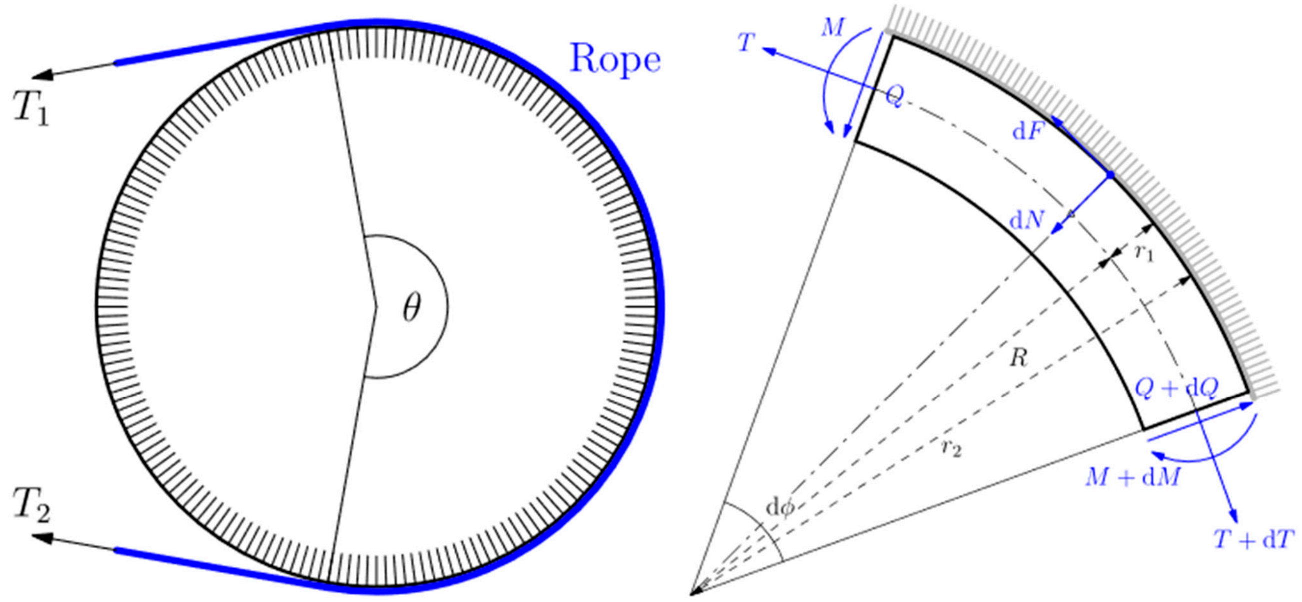

Figure S1. Capstan problem showing the classical case of rope being secured around a circular bollard (left) and our case with an implant being inserted inside of a spiral structure (right).

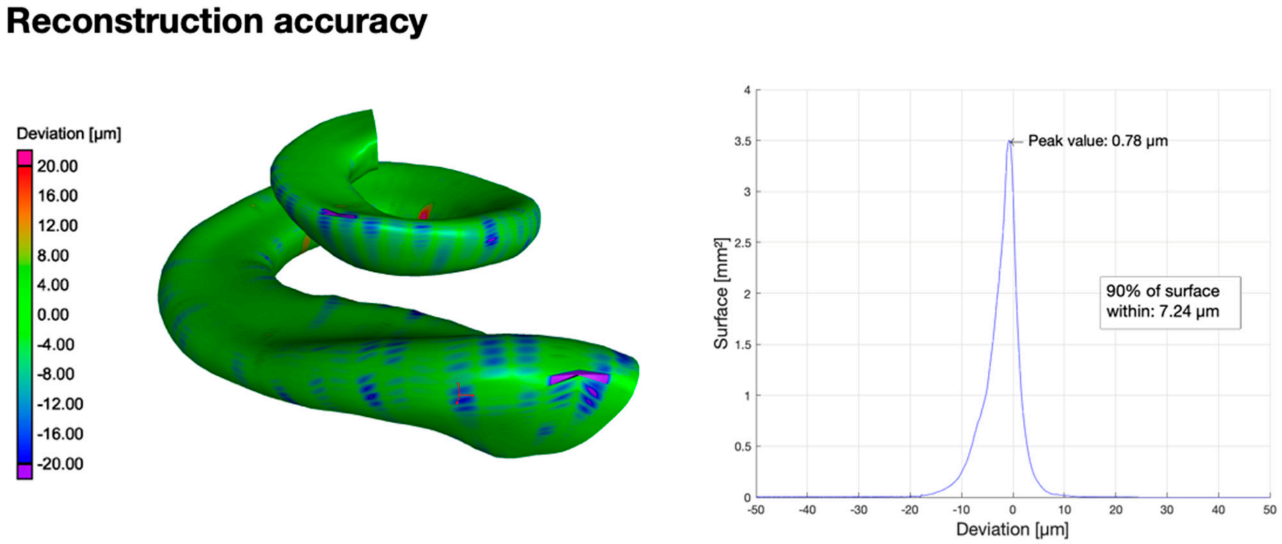

Figure S2. Accuracy of reconstructed models from shape manipulation using nominal–actual analysis. The heat map represents the overlay of ST reconstructed from cross-section lofting with the original ST segmentation mesh demonstrating good overall shape

preservation (left). The histogram manifests the deviation of 90% of the surface within 7.24 μm which highlights relatively low surface deviation when considering the low number of cross-sections (80 included in this example) relative to the original mesh (right).

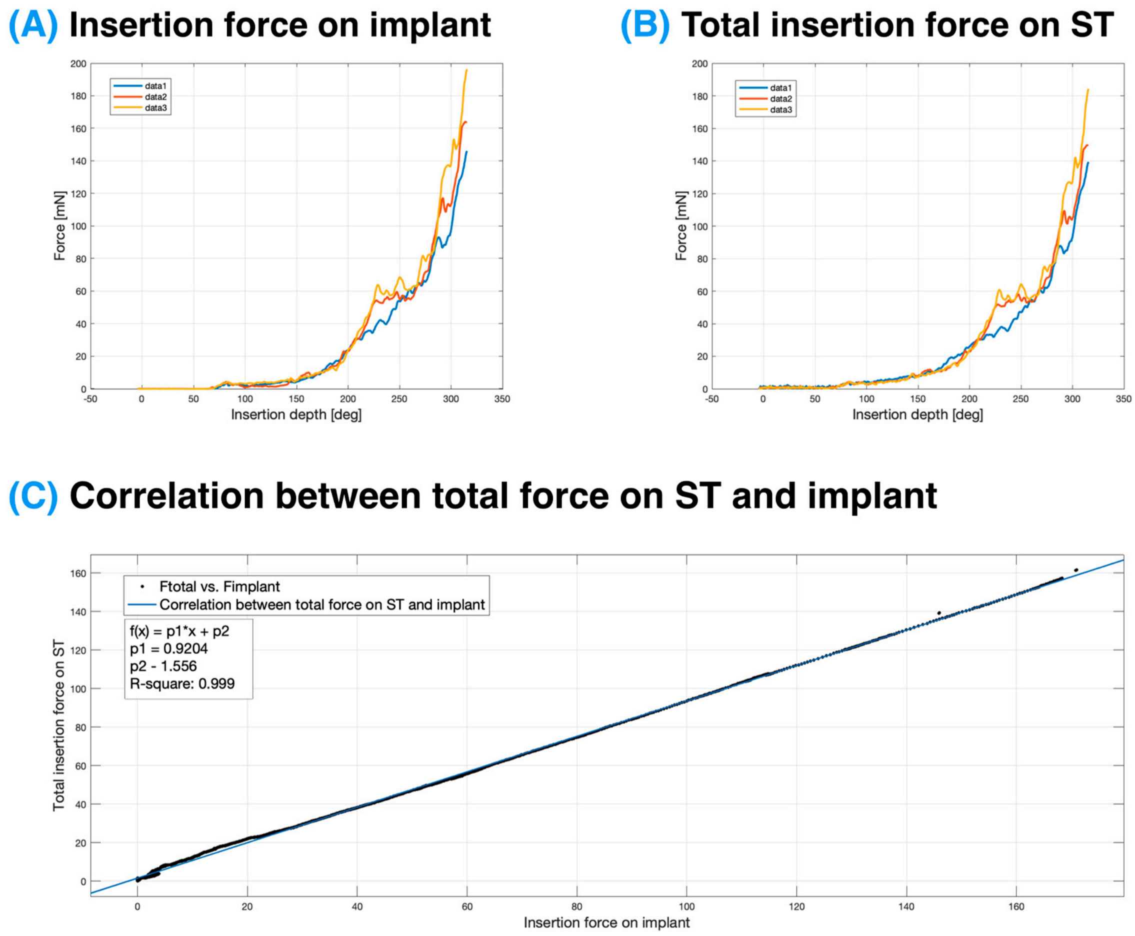

Figure S3. Cross-validation of insertion force measurements. (A) and (B) show three measurements of insertion forces on the implant and ST model measured by the one-axis sensor and the six-axis sensor, respectively. The total insertion force was calculated following equation:

$F_{Total} = \sqrt[2]{F_x^2 + F_y^2 + F_z^2}$. (C) illustrates the correlation between measurements with one-axis and six-axis sensors with an $R^2 = 0.999$.

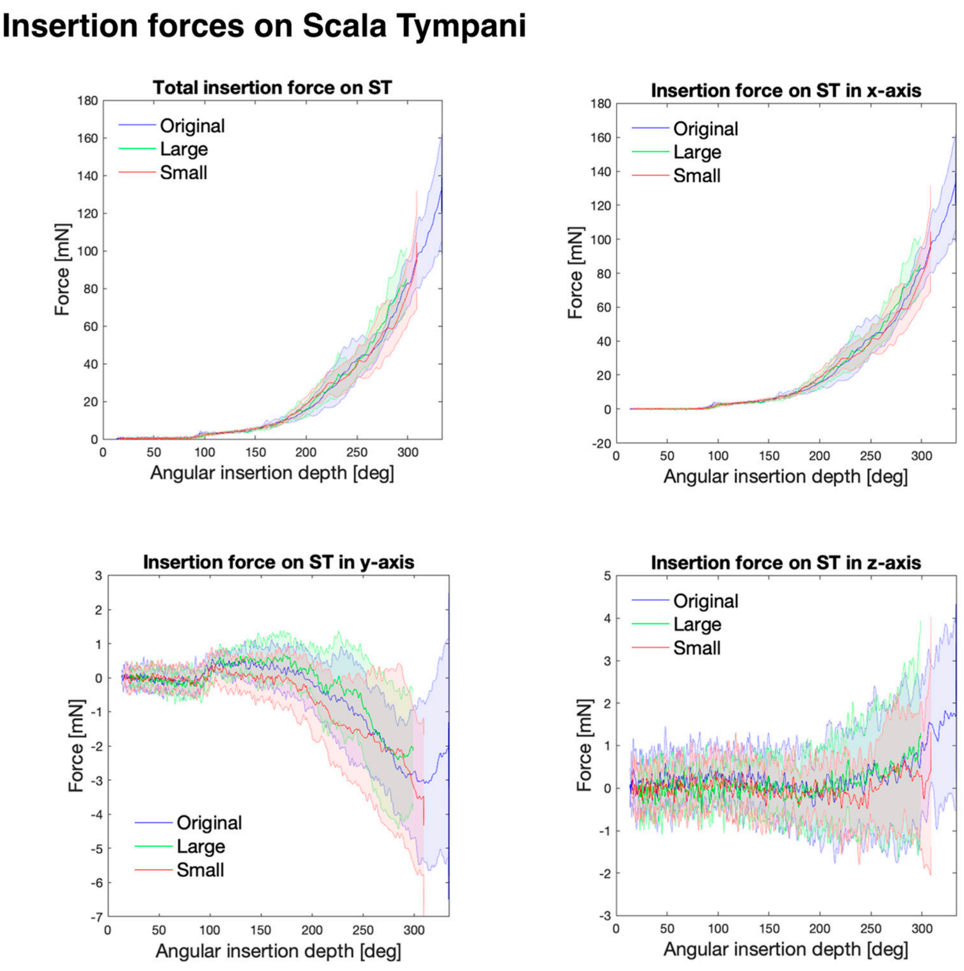

Figure S4. Total insertion forces measured on ST model (top-left) consisted of three components: insertion forces in x-axis (top-right), y-axis (bottom-left), and z-axis (bottom-right). The total insertion force was calculated following equation: $F_{Total} = \sqrt[2]{F_x^2 + F_y^2 + F_z^2}$. Insertion forces measured on the "original" (no alteration of size or shape), the "large" (110% scale of the original model volume), and the "small" (90% scale of the original model volume) model overlay once normalised for a correct angular insertion depth. Mean and standard deviation (n = 10) are highlighted by solid line and shaded area, respectively.

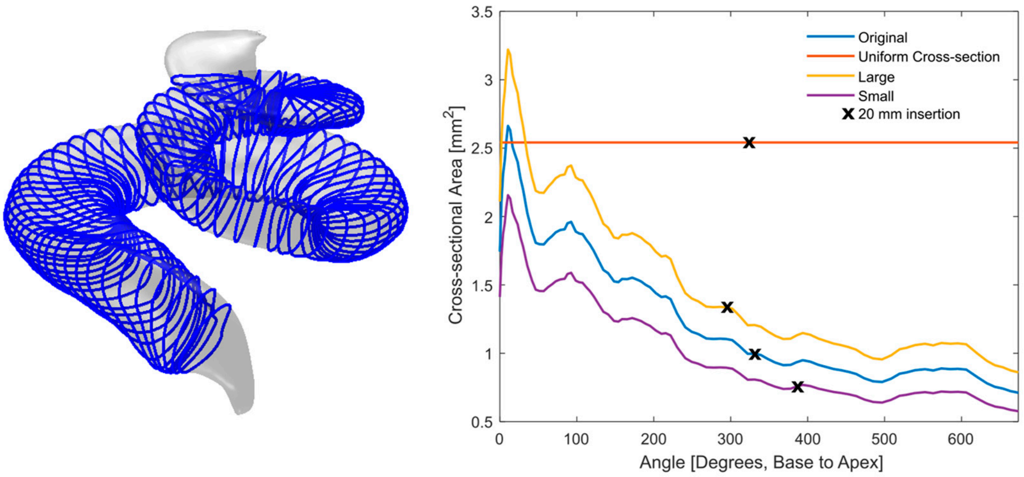

Figure S5. Cross-sectional area of selected ST models. Representation of the cross-sections on the "original" model (left) and quantification of the cross-sectional area along the ST spiral (right). Black cross displays 20 mm insertion distance of CI and its corresponding angular insertion depth.

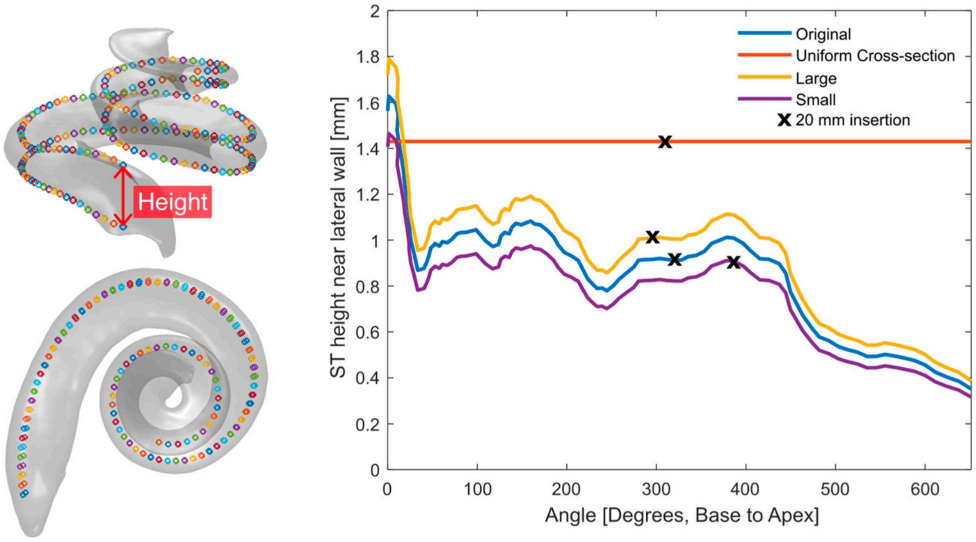

Figure S6. Height of selected ST models near the lateral wall. Representation of the points used to determine the height along the lateral wall (left) and quantification of the height in different models (right). Black cross represents 20 mm insertion distance with CI and its corresponding angular insertion depth in selected models.

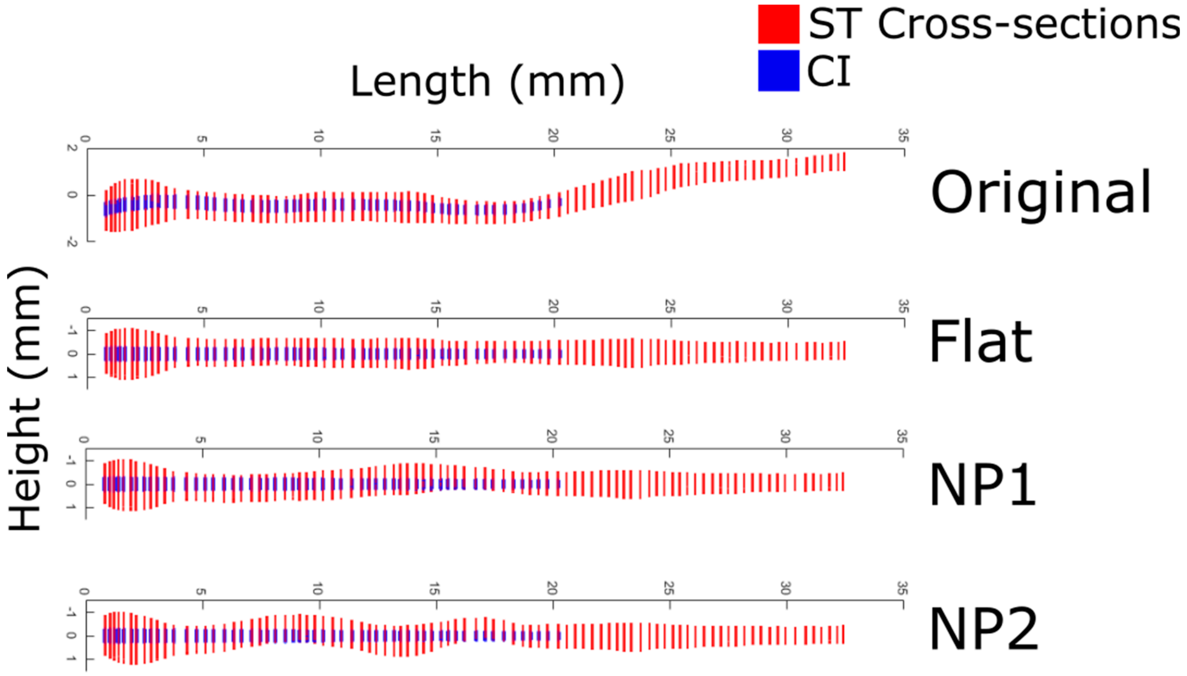

Figure S7. Size demonstration of CI (blue) and scala tympani (red) with cross-sections aligned into a straight line for the "original", "flat", and artificial non-planarity models "NP1" and "NP2".

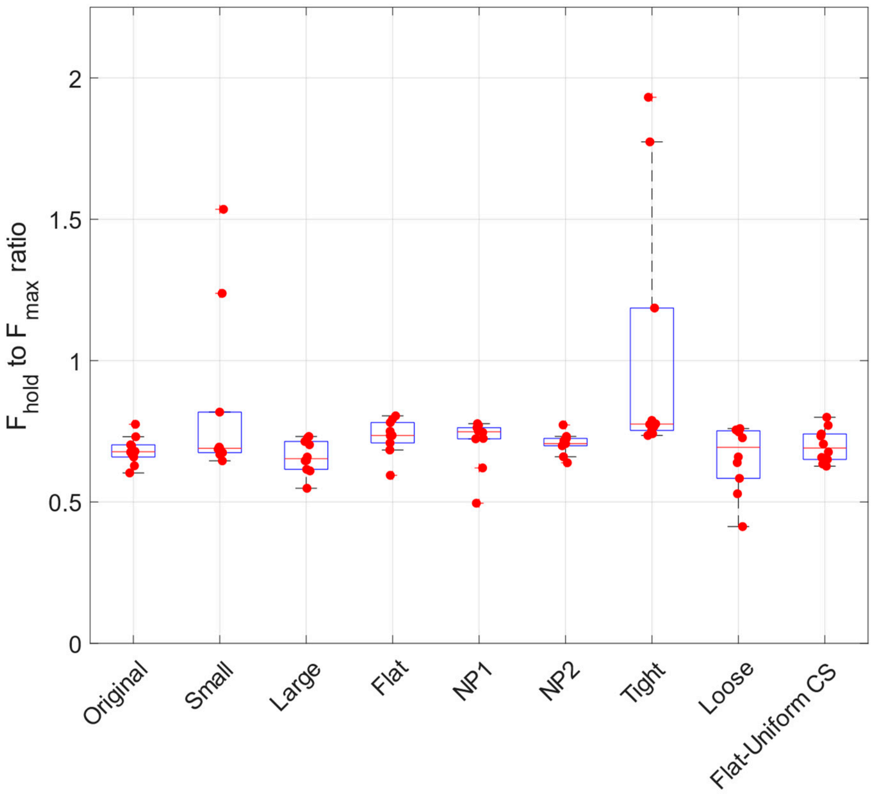

Figure S8. Ratio of relaxation for when CI held static post insertion (F_{hold}) to the maximal insertion force during insertion (F_{max}) for all models. Note that "small" and "tight" models were not fully inserted and have significant outliers and therefore are not comparable to other conditions.

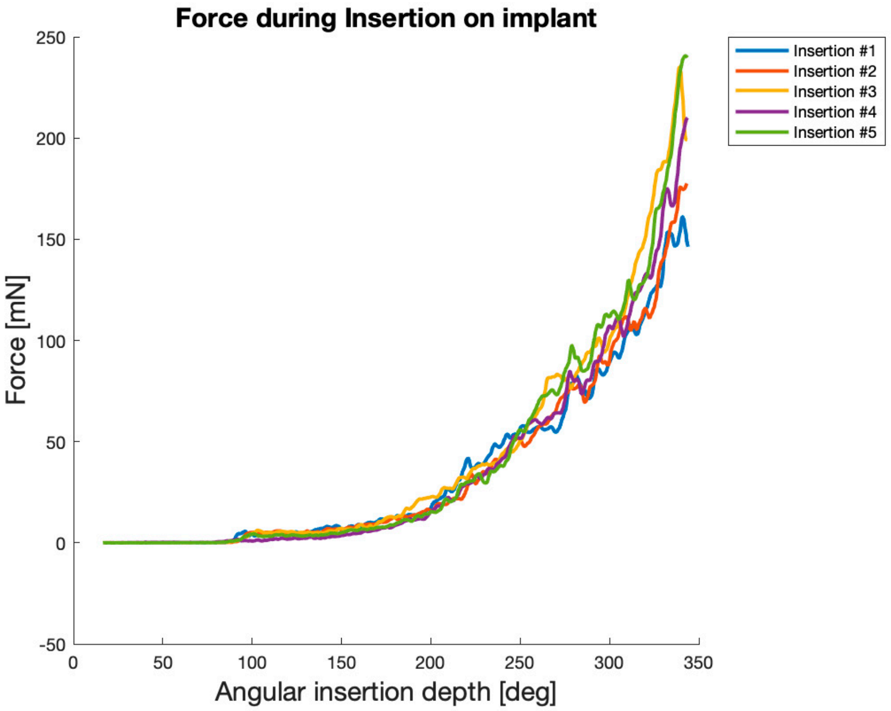

Figure S9. Example of the force exerted on the CI with 5 repeated insertions shows no trend indicating a significant change in implant intensity over repeated insertions. Note, plot displays the “flat” condition which was randomly sampled throughout all 45+ measurements with this implant in the study.

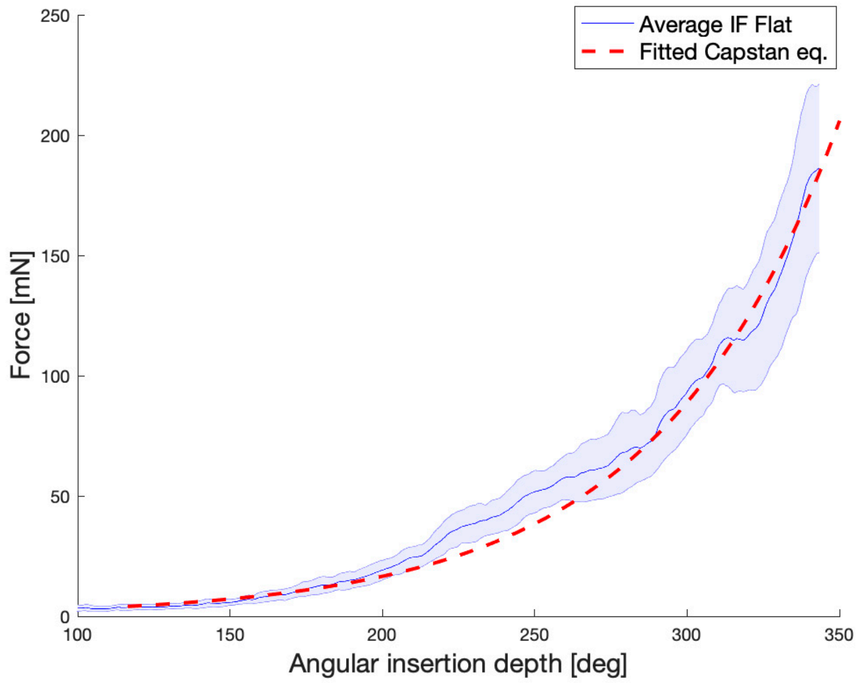

Figure S10. Representation of the fitted “flat” average insertion force profile using the Capstan equation. The solid line and the shaded area illustrate mean and standard deviation of n = 10 insertion, respectively. Red dash line shows fitted Capstan equation.

S.1. Capstan Model Equilibrium Conditions

The equilibrium conditions for the infinitesimal body section when $\mathrm{d}\phi \to 0$ in Figure S1 (right) can be given by balancing forces parallel and normal to the fibre as well as the sum of the moments around the centre of the fibre section.

$$\begin{aligned} dT + Q\mathrm{d}\phi - \mathrm{d}F = 0 \\ \mathrm{d}Q - T\mathrm{d}\phi - \mathrm{d}N = 0 \\ \mathrm{d}M - QR\mathrm{d}\phi - r\mathrm{d}F = 0 \end{aligned} \quad (S1)$$

Table S1. Summary of the mean R2 error of the force profile fitting to the exponential Capstan model, determined exponential coefficient μ′, and *p*-value compared to control within the experiment. n = 10 replicates combined over N = 2 implants; STD—standard deviation.

Experiment	Sample	R^2 Error of Fitting (Mean ± STD)	Exponential Coefficient μ′ (Mean ± STD)	p-Value
Volume scaling	Original	0.97 ± 0.011	0.017 ± 0.0008	Control
	Large	0.96 ± 0.035	0.018 ± 0.0011	0.187
	Small	0.96 ± 0.038	0.017 ± 0.0013	0.997
Manipulation of ST vertical trajectory	Original	0.97 ± 0.011	0.017 ± 0.0008	0.986
	Flat	0.97 ± 0.023	0.017 ± 0.0010	Control
	NP1	0.96 ± 0.027	0.017 ± 0.0007	0.994
	NP2	0.94 ± 0.044	0.016 ± 0.0008	0.01
Manipulation of ST curvature	Flat	0.97 ± 0.012	0.017 ± 0.0008	Control
	Flat—loose	0.96 ± 0.018	0.017 ± 0.0004	0.972
	Flat—tight	0.98 ± 0.014	0.018 ± 0.0009	0.201
Manipulation of ST cross-sectional area	Flat	0.97 ± 0.013	0.017 ± 0.0008	Control
	Flat—uniform CS	0.98 ± 0.006	0.016 ± 0.0011	0.315
